# Antimicrobial Resistance Patterns of Outpatient *Staphylococcus aureus* Isolates

**DOI:** 10.1001/jamanetworkopen.2024.17199

**Published:** 2024-06-14

**Authors:** Margaret Carrel, Matthew Smith, Qianyi Shi, Shinya Hasegawa, Gosia S. Clore, Eli N. Perencevich, Michihiko Goto

**Affiliations:** 1 Department of Geographical and Sustainability Sciences, University of Iowa, Iowa City; 2 Department of Internal Medicine, University of Iowa, Iowa City; 3 Center for Access & Delivery Research and Evaluation, Iowa City Veterans Affairs Health Care System, Iowa City, Iowa; 4 Associate Editor, *JAMA Network Open*

## Abstract

**Importance:**

Oral non–β-lactam antibiotics are commonly used for empirical therapy of *Staphylococcus aureus* infections, especially in outpatient settings. However, little is known about potential geographic heterogeneity and temporal trends in the prevalence of *S aureus* resistance to non–β-lactams in the US.

**Objective:**

To characterize the spatiotemporal trends of resistance to non–β-lactam antibiotics among community-onset *S aureus* infections, including regional variation in resistance rates and geographical heterogeneity in multidrug resistance.

**Design, Setting, and Participants:**

This cross-sectional study used data from Veterans Health Administration clinics collected from adult outpatients with *S aureus* infection in the conterminous 48 states and Washington, DC, from January 1, 2010, to December 31, 2019. Data were analyzed from January to November 2023.

**Exposures:**

Resistance to lincosamides (clindamycin), tetracyclines, sulfonamides (trimethoprim-sulfamethoxazole [TMP-SMX]), and macrolides.

**Main Outcomes and Measures:**

Spatiotemporal variation of *S aureus* resistance to these 4 classes of non–β-lactam antibiotics, stratified by methicillin-resistant *S aureus* (MRSA) and methicillin-sensitive *S aureus* (MSSA), and subdivided by regions of the US (Northeast, Midwest, South, and West). Trend tests and bivariate mapping were used to determine significant changes in resistant proportions over time and identify counties where rates of resistance to multiple non–β-lactams were high.

**Results:**

A total of 382 149 *S aureus* isolates from 268 214 unique outpatients (mean [SD] age, 63.4 [14.8] years; 252 910 males [94.29%]) were analyzed. There was a decrease in the proportion of MRSA nationwide, from 53.6% in 2010 to 38.8% in 2019. Among MRSA isolates, we observed a significant increase in tetracycline resistance (from 3.6% in 2010 to 12.8% in 2019; *P* for trend < .001) and TMP-SMX resistance (from 2.6% in 2010 to 9.2% in 2019; *P* for trend < .001), modest and not significant increases in clindamycin resistance (from 24.2% in 2010 to 30.6% in 2019; *P* for trend = .34), and a significant decrease in macrolide resistance (from 73.5% in 2010 to 60.2% in 2019; *P* for trend < .001). Among MSSA isolates, significant upward trends in clindamycin, tetracyclines, and TMP-SMX resistance were observed. For example, tetracycline resistance increased from 3.7% in 2010 to 9.1% in 2019 (*P* for trend < .001). Regional stratification over time showed that the Northeast had slightly higher rates of clindamycin resistance but lower rates of tetracycline resistance, while the South had notably higher rates of resistance to tetracyclines and TMP-SMX, particularly among MRSA isolates. Bivariate mapping at the county scale did not indicate clear regional patterns of shared high levels of resistance to the 4 classes of antimicrobials studied.

**Conclusions and Relevance:**

In this study of outpatient *S aureus* isolates, MRSA became less common over the 10-year period, and MRSA isolates were increasingly resistant to tetracyclines and TMP-SMX. Geographic analysis indicated no spatial overlap in counties with high rates of resistance to both tetracyclines and TMP-SMX. Examining the regional spatial variation of antibiotic resistance can inform empirical therapy recommendations and help to understand the evolution of *S aureus* antibiotic resistance mechanisms.

## Key Points

**Question:**

What are the spatiotemporal trends in *Staphylococcus aureus* resistance to non–β-lactam antibiotics in the US?

**Findings:**

In this cross-sectional study of 382 149 *S aureus* isolates, significant increases in resistance to tetracyclines and trimethoprim-sulfamethoxazole were observed over a 10-year period, particularly in the southern US, although multidrug co-occurrence of resistance was rare.

**Meaning:**

Findings of this cross-sectional study suggest that health care professionals may need to tailor their prescribing behaviors in outpatient settings based on local trends in *S aureus* resistance.

## Teaser

This cross-sectional study examines spatiotemporal trends in *Staphylococcus aureus* resistance to non–β-lactam antibiotics in the US from 2010 to 2019.

## Introduction

*Staphylococcus aureus* is a major cause of infections in both inpatient and outpatient settings.^1,2,3,4,5^The emergence of methicillin-resistant *S aureus* (MRSA), particularly in communities in the 1990s, raised alarms and resulted in both greater surveillance of MRSA and implementation of strategies to prevent MRSA transmission in clinical settings.^1,4,6,7,8,9,10^ While there is evidence of a declining proportion of MRSA in the US, the possibility of *S aureus* resistance to β-lactam antibiotics continues to influence empirical prescribing behaviors, with health care professionals often choosing to use non–β-lactam oral antibiotics, such as clindamycin, tetracyclines, and trimethoprim-sulfamethoxazole (TMP-SMX) as the first choice for empirical treatment.^1,2,11,12,13,14,15,16^

Prior research has indicated widely varying resistance rates among *S aureus* to clindamycin, tetracyclines, and TMP-SMX.^17,18,19,20,21,22,23^ There is difficulty in assessing resistance patterns in space and time, and any potential spatial correlation among resistances to different classes of non–β-lactam antimicrobials, because of limited national surveillance of drug resistance patterns among *S aureus* in the US. Surveillance sites for the Centers for Disease Control and Prevention Emerging Infections Program Healthcare-Associated Infections Community Interface were focused on invasive MRSA infections from 2004 to 2014 and only began tracking invasive methicillin-susceptible *S aureus* (MSSA) infections in 2016.^5,24^ Other shortcomings of these surveillance programs included a lack of assessment of *S aureus* resistance to non–β-lactam antimicrobials and noninvasive infections. In addition, these national surveillance sites are almost exclusively in metropolitan areas, potentially missing important trends taking place in rural areas of the US.^25^ Other studies of trends in antimicrobial resistance among *S aureus* isolates focused primarily on large health care settings or inpatient populations rather than smaller community clinics serving outpatients.^26,27,28^ Thus, knowledge about trends in antimicrobial resistance patterns for *S aureus* other than MRSA in community settings is limited.

The Veterans Health Administration (VHA) is the only nationwide integrated provider of health care in the US and has curated microbiological data from its electronic medical record system for all *S aureus* isolates obtained within outpatient settings, including antimicrobial susceptibility data and geocoded residential addresses of source patients. In this study, we used VHA data from 2010 to 2019 to describe the overall trends of resistance to non–β-lactam antimicrobials among *S aureus* infections in outpatients, regional variations in resistance rates, and geographical heterogeneity in multidrug resistance.

## Methods

This cross-sectional study was approved by the institutional review board at the University of Iowa and the Research and Development Committee at the Iowa City Veterans Affairs Health Care System with a waiver of informed consent because the study was a retrospective analysis of health records with no direct contact with patients. This study followed the Strengthening the Reporting of Observational Studies in Epidemiology (STROBE) reporting guideline.

Data from all *S aureus* clinical specimens from outpatient settings in the VHA from January 1, 2010, to December 31, 2019, were extracted from the VHA’s Corporate Data Warehouse. Specimens were excluded if they were obtained from a patient younger than 18 years, if they were missing geographic information, if they were obtained from a patient living outside of the conterminous 48 states or Washington, DC, or if they were obtained more than 48 hours after admission to institutionalized settings (eg, acute care, nursing home) and less than 72 hours after discharge. Specimens lacking microbiological susceptibility test results were also excluded. To avoid overestimation of trends based on persistent or recurrent infections or multiple specimens taken from a single infection, only the first specimen per patient per month was included.

Microbiology reports were used to assign MRSA or MSSA status and to assess resistance or susceptibility to 4 antimicrobial classes commonly used in outpatient empirical therapy settings: lincosamides (clindamycin), tetracyclines (tetracycline, doxycycline, and minocycline), sulfonamides (TMP-SMX), and macrolides (erythromycin, clarithromycin, and azithromycin). We included macrolides in this analysis because they have been extensively used to treat *S aureus* infection in the past, although current guidelines do not list them as preferred agents. Methicillin resistance was defined as an isolate having resistance to β-lactam agents with antistaphylococcal activity, including intermediate resistance, to any cephalosporin (except for ceftaroline), antistaphylococcal penicillin, or carbapenem. In practice, most clinical microbiology laboratories within the VHA system use susceptibility to first-generation cephalosporin or cefoxitin to determine MRSA vs MSSA status rather than phenotypic susceptibility testing for methicillin or oxacillin.

### Statistical Analysis

The monthly frequency of *S aureus* isolates obtained from patients from 2010 to 2019 was calculated, as was the changing prevalence of MRSA among *S aureus* isolates over time. The prevalence of MRSA was determined as the proportion of isolates defined as MRSA out of all *S aureus* isolates meeting inclusion criteria in each month. Isolates were assigned to 1 of 4 US Census regions (Northeast, South, Midwest, or West) based on the county of the patient’s residential address, and the monthly prevalence of MRSA by region was also assessed.

To understand spatiotemporal variation in antibiotic resistance among *S aureus* isolates, the monthly prevalence of resistance to the 4 antimicrobial classes stratified by MRSA and MSSA status was calculated overall and per region for each month of the study. Trend analysis using the 2-tailed Mann-Kendall test, with a sieve-bootstrap method used for potentially autocorrelated data, examined the potential for significant, monotonic trends in resistance over time stratified by MRSA or MSSA status and antimicrobial class.^29^ Significance was determined as *P* < .05.

To explore the potential for regional patterns of resistance to multiple classes of antimicrobials in MRSA and MSSA isolates, the Pearson correlation coefficient was used to detect a direct or inverse association between the prevalence of different resistance types. Correlation was calculated annually (2010-2019) for all isolates in the dataset (ie, overall) and by region to test, for instance, if the correlation between the prevalence of resistance to tetracyclines was correlated with the prevalence of resistance to TMP-SMX in the West in 2010. Significance was determined by 2-tailed *P* < .05.

Bivariate descriptive mapping at the county level was used to visualize how high and low rates of resistance co-occur among isolates for antimicrobial classes that showed evidence of increased rates over time. Proportions of MRSA and MSSA isolates that were resistant to either tetracyclines or TMP-SMX were calculated for each US county in 2019 based on patients’ residential addresses. The proportions of MRSA and MSSA isolates that were resistant to each of these 2 drug classes were then classified into 4 categories (0%-10%, 11%-20%, 21%-50%, and 51%-100%), and bivariate maps were created to show membership in resistance categories for both tetracyclines and TMP-SMX. Cutoffs were chosen based on clinician feedback about acceptable empirical therapy failure rates.^30^

All statistical analysis was done in RStudio, version 2022.07.2.576 (R Foundation for Statistical Computing) using the funtimes and ggcorrplot packages.^31^ Bivariate mapping was done in ArcGIS Pro, version 3.0 (Esri). Data were analyzed from January to November 2023.

## Results

**Figure 1.**
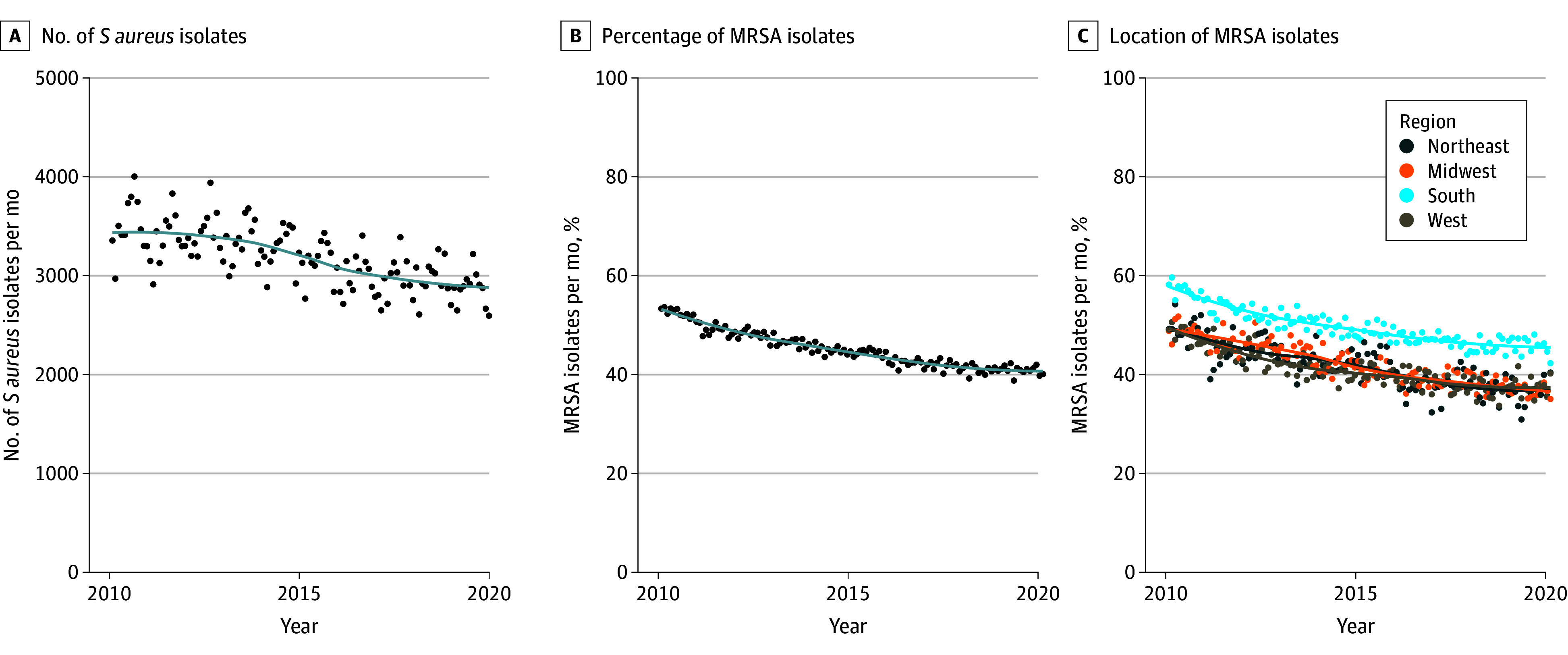
Patterns of *Staphylococcus aureus* Isolates From 2010 to 2019 MRSA indicates methicillin-resistant *S aureus.*

**Figure 2.**
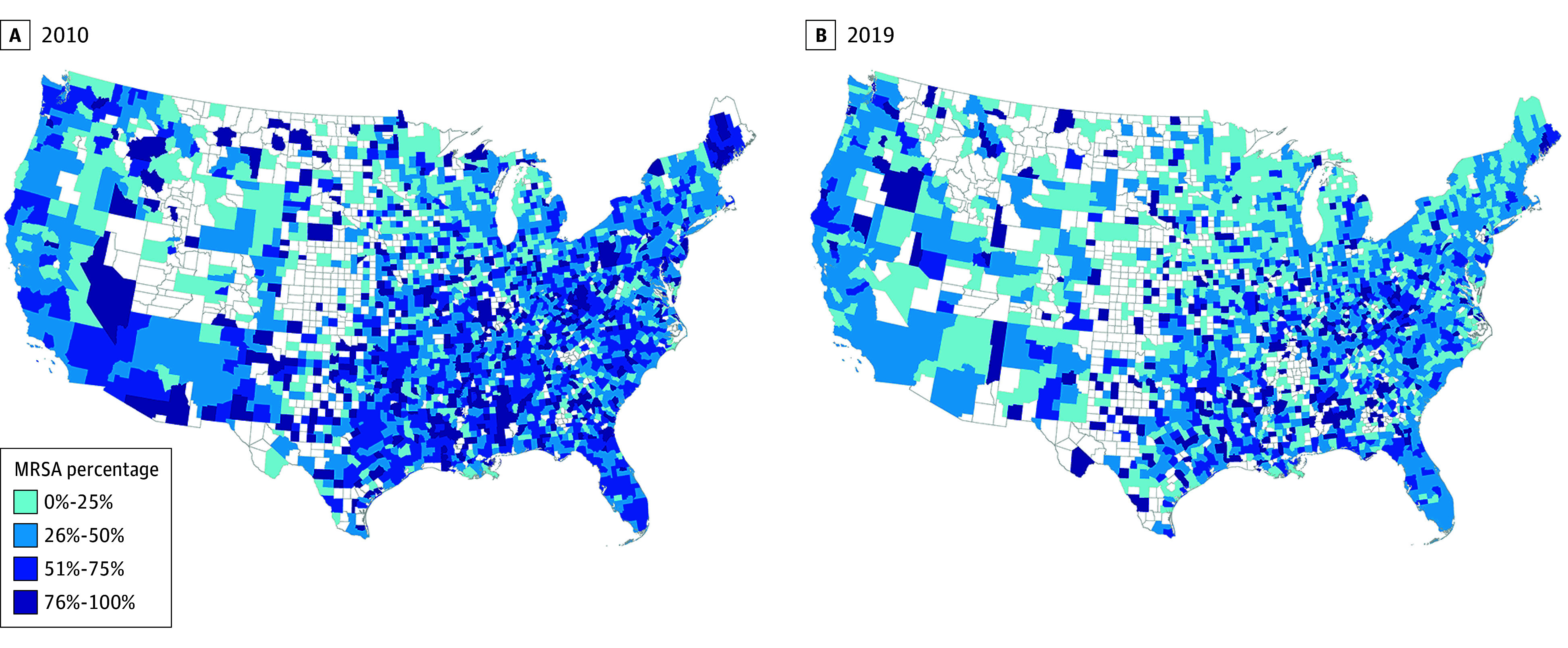
*Staphylococcus aureus* Isolates Classified as Methicillin Resistant (MRSA) in US Counties, 2010 and 2019

A total of 382 149 *S aureus* isolates from 268 214 unique outpatients between 2010 and 2019 were included in the study. Patients included 252 910 males (94.3%) and 15 304 females (5.7%); mean (SD) age was 63.4 (14.8) years, with most patients (224 779 [83.8%]) aged 50 years and older (eTable in Supplement 1). The number of *S aureus* specimens obtained in VHA outpatient settings decreased over the study period, from a high of 42 003 in 2010 to a low of 34 437 in 2019 (Figure 1A), although there was a high degree of monthly variation (eFigure 1 in Supplement 1). *S aureus* specimens were most commonly obtained in the summer months across all years. Of the 382 149 specimens included in the study, 173 118 (45.3%) were classified as MRSA. The percentage of isolates that were MRSA also decreased over the study period, from a high of 53.6% in 2010 to a low of 38.8% in 2019 (Figure 1B). This decrease was observed in all regions, although the highest percentage of MRSA isolates remained consistently in the South (Figure 1C and Figure 2).

**Figure 3.**
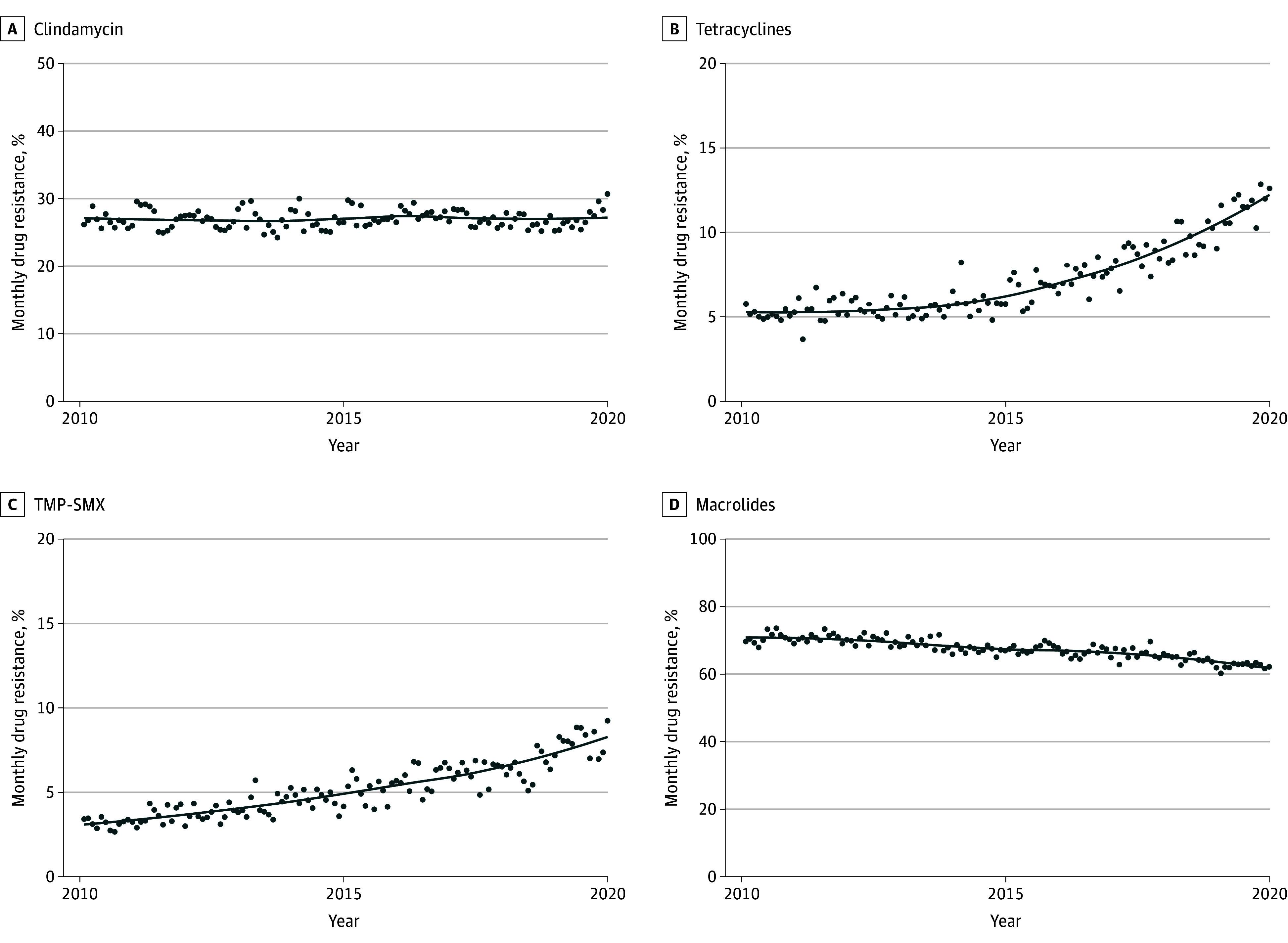
Prevalence Among Methicillin-Resistant *Staphylococcus aureus* (MRSA) Isolates of Resistance to Clindamycin, Tetracycline, Trimethoprim-Sulfamethoxazole (TMP-SMX), and Macrolides From 2010 to 2019 No significant trend was found in the prevalence of resistance to clindamycin (A); however, there were significantly increased trends in the prevalence of resistance to tetracycline (B) and TMP-SMX (C). A significantly decreased trend in the prevalence of resistance to macrolides was found (D).

When stratified by MRSA vs MSSA, antimicrobial resistance rates varied over the study period. Among MRSA isolates, clindamycin resistance varied from 24.2% to 30.6%, increasing over the study period, but not significantly so, while clindamycin resistance remained below 20% among MSSA isolates (Figure 3A and eFigure 2A in Supplement 1). Higher levels of tetracyclines and TMP-SMX resistance were observed in MRSA isolates compared with MSSA isolates during the study period, with tetracycline resistance rising from 3.6% in 2010 to 12.8% in 2019 (*P* for trend < .001) and TMP-SMX resistance rising from 2.6% in 2010 to 9.2% in 2019 (*P* for trend < .001) (Figure 3B and C). Resistance to macrolides occurred at higher levels in MRSA isolates than in MSSA isolates, although a reduction in resistance from 73.5% in 2010 to 60.2% in 2019 (*P* for trend < .001) among MRSA isolates was observed (Figure 3D; eFigure 2D in Supplement 1). As for MSSA specimens, significant increases in the levels of resistance to clindamycin (from 13.1% in 2010 to 18.7% in 2019; *P* for trend < .001), tetracyclines (from 3.7% in 2010 to 9.1% in 2019; *P* for trend < .001), and TMP-SMX (from 0.9% in 2010 to 2.7% in 2019; *P* for trend < .001) were found, with no significant trend in macrolide resistance (from 25.4% in 2010 to 28.9% in 2019; *P* for trend = .35).

**Figure 4.**
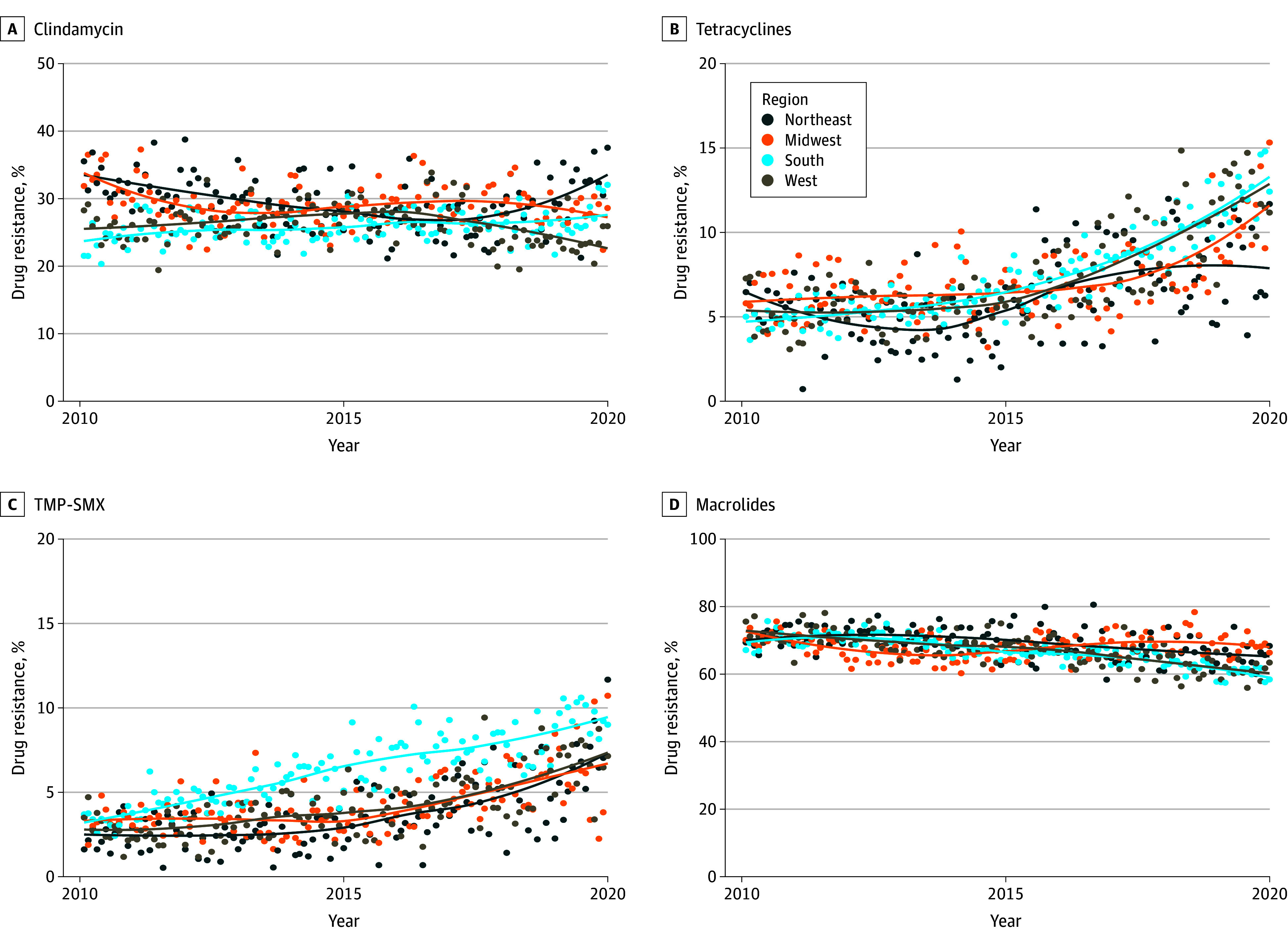
Regional Prevalence Among Methicillin-Resistant *Staphylococcus aureus* (MRSA) Isolates of Resistance to Clindamycin, Tetracycline, Trimethoprim-Sulfamethoxazole (TMP-SMX), and Macrolides From 2010 to 2019

Stratification by region indicated that much of the increase in tetracyclines-resistant MRSA was attributable to isolates in the western and southern regions of the US (Figure 4B). In addition, MRSA isolates from the South exhibited the highest TMP-SMX resistance rates (Figure 4C). Regional patterns were not as clear among MSSA isolates (eFigure 3 in Supplement 1).

Pearson correlation coefficient analysis of MRSA and MSSA isolates over time and by region indicated a direct and significant correlation between clindamycin and macrolide resistance in all years and regions for both MRSA and MSSA, although correlations were stronger for MSSA (eFigure 4 in Supplement 1). For example, among MSSA isolates in the Northeast, clindamycin and macrolide resistance had correlation coefficients greater than 0.75 in all years of the study. A direct and significant correlation between tetracyclines and TMP-SMX resistance was sporadically observed across regions and time, particularly for MRSA. For example, this correlation was observed in the early years of the study and particularly in the Northeast and South. Insignificant or inverse correlations for macrolides and TMP-SMX were found. This inverse correlation was most apparent in the early years of the study.

**Figure 5.**
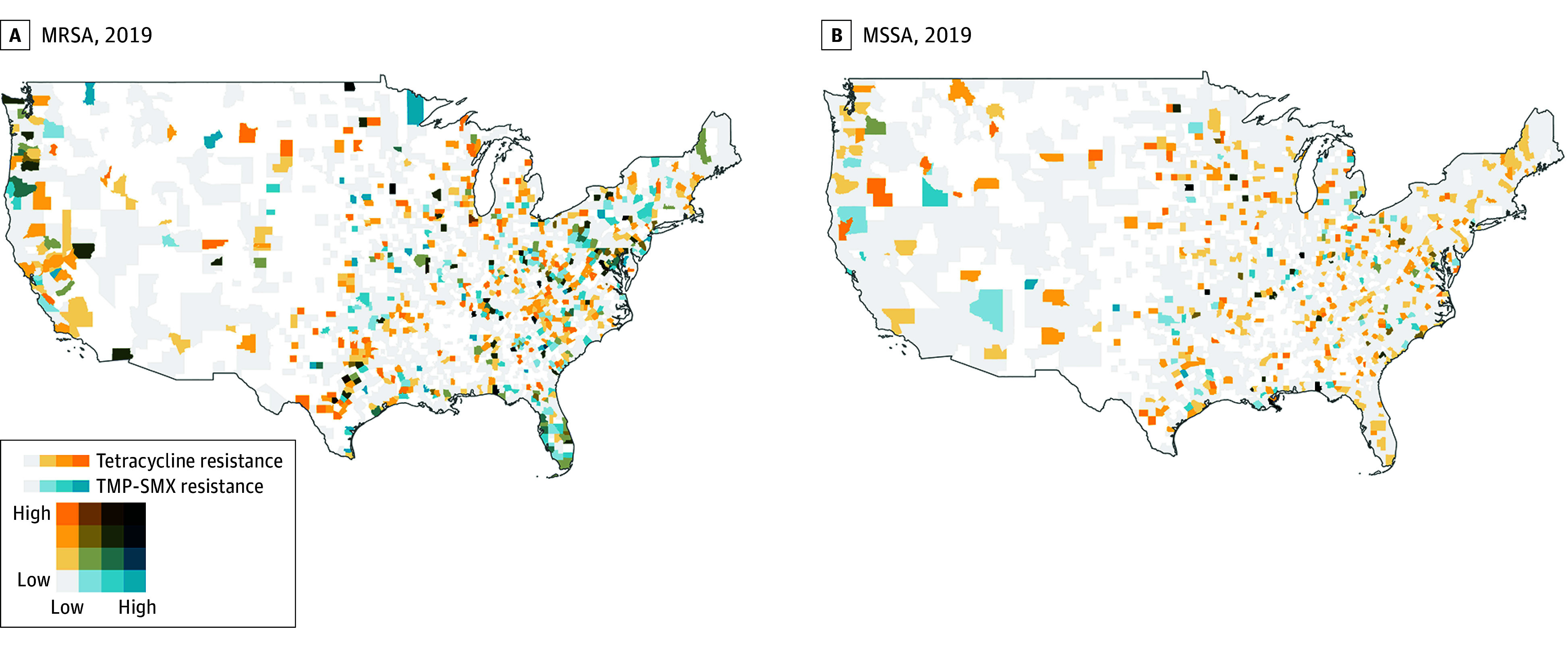
Bivariate Map of Resistance to Tetracyclines and Trimethoprim-Sulfamethoxazole (TMP-SMX) in Methicillin-Resistant *Staphylococcus aureus* (MRSA) and Methicillin-Susceptible *S aureus* (MSSA) Isolates in US Counties in 2019 Resistance was categorized as 0%-10% (low), 11%-20%, 21%-50%, or 51%-100% (high). Counties in white had no *S aureus* samples from outpatients in 2019.

Trends in resistance were highest for tetracyclines and TMP-SMX resistance in MRSA isolates, and significant increases in resistance to these classes were also observed in MSSA isolates. Bivariate mapping of tetracyclines and TMP-SMX resistance in counties among MRSA and MSSA isolates in 2019, when rates of resistance were greatest, indicated only a few sporadic counties with rates of resistance to both tetracyclines and TMP-SMX greater than 50% (Figure 5). For MRSA isolates, the highest levels of co-occurrence of resistance to tetracyclines and TMP-SMX were observed in Appalachia and the Upper Midwest, and for MSSA isolates, the highest levels of co-occurrence of resistance to tetracyclines and TMP-SMX were observed along the Gulf Coast. Many noncontiguous counties in all 4 US Census regions had rates of resistance to both tetracyclines and TMP-SMX above 10%.

## Discussion

In this study of data from a nationwide cohort of specimens from outpatients, the prevalence of both *S aureus* and MRSA declined from 2010 to 2019 in all regions of the conterminous US and Washington, DC, consistent with prior studies.^1,2,12,17^ Rates of MRSA were highest in the South over the duration of the study period. Prior work has suggested that the combination of sociodemographic factors (eg, crowding, poverty), climate factors (eg, heat, humidity), and antibiotic prescribing patterns have contributed to consistently higher rates of antimicrobial resistance in the South.^32,33,34,35,36^ Climate factors and changing behavior in patient population also likely play a role in the seasonality of *S aureus* infections observed in the patient cohort.^36,37,38^

Levels of antimicrobial resistance to non–β-lactams varied between MRSA and MSSA isolates. While the rates of tetracycline resistance at the beginning of the study period were similar between MRSA and MSSA, the rate of tetracycline resistance increased more in MRSA isolates by 2019, although both exhibited a statistically significant increases. Similarly, while both MRSA and MSSA isolates had significant increased trends in resistance to TMP-SMX, rates were consistently higher in MRSA isolates across the study period. When stratified regionally, the South was again the site of the greatest rates of resistance to tetracyclines and TMP-SMX. Increased resistance to TMP-SMX in MRSA has also been observed in nationwide inpatient datasets in the US, although regional stratification indicated the highest rates in the West and Midwest rather than the South.^20^

Despite a significantly increased trend in clindamycin resistance in MSSA isolates and a significantly decreased trend in macrolide resistance in MRSA isolates, the overall rates of clindamycin and macrolide resistance were much higher in MRSA than MSSA isolates. Higher clindamycin resistance in MRSA than MSSA has previously been observed in multiple patient populations, including a nationwide pediatric cohort.^17,39,40^ Correlation analysis by region and year indicated a direct correlation between clindamycin and macrolide resistance. Correlation between clindamycin and macrolide resistance may be underestimated in the analysis due to undetected inducible clindamycin resistance from mechanisms associated with macrolide resistance.^41^ In contrast, while the rates of tetracyclines and TMP-SMX resistance significantly increased, particularly in MRSA isolates, little spatial correlation was observed between the 2 resistance types. Mapping rates of tetracyclines and TMP-SMX resistance showed only a few counties with rates of resistance to both drug classes greater than 50%, but those maps also identified many noncontiguous counties with rates of resistance to both classes at levels above which clinicians may alter empirical prescribing behavior.^30^ Thus, while regionally there are increases in resistance, they were not happening simultaneously in all counties within the region, which suggests greater need for spatially explicit reporting of resistance rates.

### Limitations

Although the VHA is the only nationwide integrated provider of health care in the US, the population it serves skews older and male and is not necessarily representative of the larger US patient population. While not sociodemographically representative, however, the VHA population is more spatially representative of the conterminous US than other populations used for antimicrobial resistance surveillance. The VHA serves a high number of rural residents, unlike other surveillance populations, although disaggregation by rural vs urban county was not examined in this study. Additionally, only infections among outpatients were considered in this analysis of antimicrobial resistance among community *S aureus* infections; different rates of resistance to non–β-lactam agents might be observed among inpatients with infections. The study period concluded in 2019 to avoid confounding by the COVID-19 pandemic in terms of both greater antimicrobial usage for secondary infections after coronavirus infections and avoidance of outpatient care by patients worried about contagion. Future research is needed to assess whether the antimicrobial susceptibility patterns observed prior to 2019 held after the pandemic.

## Conclusions

This cross-sectional study revealed significant changes in antimicrobial susceptibility among *S aureus* isolates from 2010 to 2019. Fewer than half of the isolates in 2019 were resistant to β-lactam antimicrobials, even in the South. However, MRSA isolates compared with MSSA isolates exhibited higher levels of resistance to all 4 antimicrobial classes analyzed, with significant increases in tetracyclines and TMP-SMX resistance. While there was little co-occurrence of very high levels of resistance to both tetracyclines and TMP-SMX in US counties, there was regional variation in rates of resistance and some regional correlation in resistance rates to the 4 classes of antimicrobials analyzed in the study. This finding suggests that clinicians, particularly in the South, should be aware of how susceptibility patterns are changing in their patient populations and spatially tailor their prescribing behaviors. Geographically specific antibiograms, particularly for outpatient facilities that serve a large patient catchment area, could better inform empirical therapy decisions, although evidence suggests that such antibiograms need to incorporate seasonality and patient-level information.^42^ Additionally, regional variation in resistance trends suggests that greater geographic representation in surveillance populations, particularly in more rural areas of the US, is needed to gain a full understanding of antimicrobial resistance among *S aureus* in community settings.

## Notes

Supplement 1. **eTable.** Demographic Characteristics of Individuals with Outpatient *S aureus* Isolates **eFigure 1.** Count of *S aureus* Isolates by Month, Stratified by Year **eFigure 2.** Prevalence Among MSSA Isolates of Resistance to Clindamycin (A), Tetracyclines (B), Trimethoprim-Sulfamethoxazole (TMP-SMX) (C), and Macrolides (D) **eFigure 3.** Regional Prevalence Among MSSA Isolates of Resistance to Clindamycin (A), Tetracyclines (B), Trimethoprim-Sulfamethoxazole (TMP-SMX) (C), and Macrolides (D) **eFigure 4.** Pearson Correlation Coefficients in MRSA and MSSA Isolates, Overall and Stratified by Year and Region, for Correlation Between Prevalence Rates of Clindamycin (C), Tetracycline (T), Trimethoprim-Sulfamethoxazole (S), and Macrolides (M) Resistance

Supplement 2.
Data Sharing Statement
