# Supplementary material for: Antimicrobial Resistance Patterns of Outpatient Staphylococcus aureus Isolates
Source: JAMA Netw Open. 2024 Jun 14;7(6):e2417199. doi: 10.1001/jamanetworkopen.2024.17199 (PMC11179135; doi:10.1001/jamanetworkopen.2024.17199)
Supplement: Supplement 1. — eTable. Demographic Characteristics of Individuals with Outpatient S aureus Isolates eFigure 1. Count of S aureus Isolates by Month, Stratified by Year eFigure 2. Prevalence Among MSSA Isolates of Resistance to Clindamycin (A), Tetracyclines (B), Trimethoprim-Sulfamethoxazole (TMP-SMX) (C), and Macrolides (D) eFigure 3. Regional Prevalence Among MSSA Isolates of Resistance to Clindamycin (A), Tetracyclines (B), Trimethoprim-Sulfamethoxazole (TMP-SMX) (C), and Macrolides (D) eFigure 4. Pearson Correlation Coefficients in MRSA and MSSA Isolates, Overall and Stratified by Year and Region, for Correlation Between Prevalence Rates of Clindamycin (C), Tetracycline (T), Trimethoprim-Sulfamethoxazole (S), and Macrolides (M) Resistance [file jamanetwopen-e2417199-s001.pdf]

Supplemental Online Content

Carrel M, Smith M, Shi Q, et al. Antimicrobial resistance patterns of outpatient *Staphylococcus aureus* Isolates in the US. *JAMA Netw Open*. 2024;7(6):e2417199. doi: 10.1001/jamanetworkopen.2024.17199

eTable. Demographic Characteristics of Individuals with Outpatient *S aureus* Isolates

eFigure 1. Count of *S aureus* Isolates by Month, Stratified by Year

eFigure 2. Prevalence Among MSSA Isolates of Resistance to Clindamycin (A), Tetracyclines (B), Trimethoprim-Sulfamethoxazole (TMP-SMX) (C), and Macrolides (D)

eFigure 3. Regional Prevalence Among MSSA Isolates of Resistance to Clindamycin (A), Tetracyclines (B), Trimethoprim-Sulfamethoxazole (TMP-SMX) (C), and Macrolides (D)

eFigure 4. Pearson Correlation Coefficients in MRSA and MSSA Isolates, Overall and Stratified by Year and Region, for Correlation Between Prevalence Rates of Clindamycin (C), Tetracycline (T), Trimethoprim-Sulfamethoxazole (S), and Macrolides (M) Resistance

This supplemental material has been provided by the authors to give readers additional information about their work.

eTable. Demographic Characteristics of Individuals with Outpatient *S aureus* Isolates

**Variable**	**N**	**Percent**
Total	268214	
Age, y		
Mean (SD)	63.4 (14.8)	
18-34	15166	5.65%
35-49	28269	10.54%
50-64	97300	36.28%
65-79	91616	34.16%
80+	35863	13.37%
Gender		
Female	15304	5.71%
Male	252910	94.29%

Age is taken at time of first entry into the cohort.

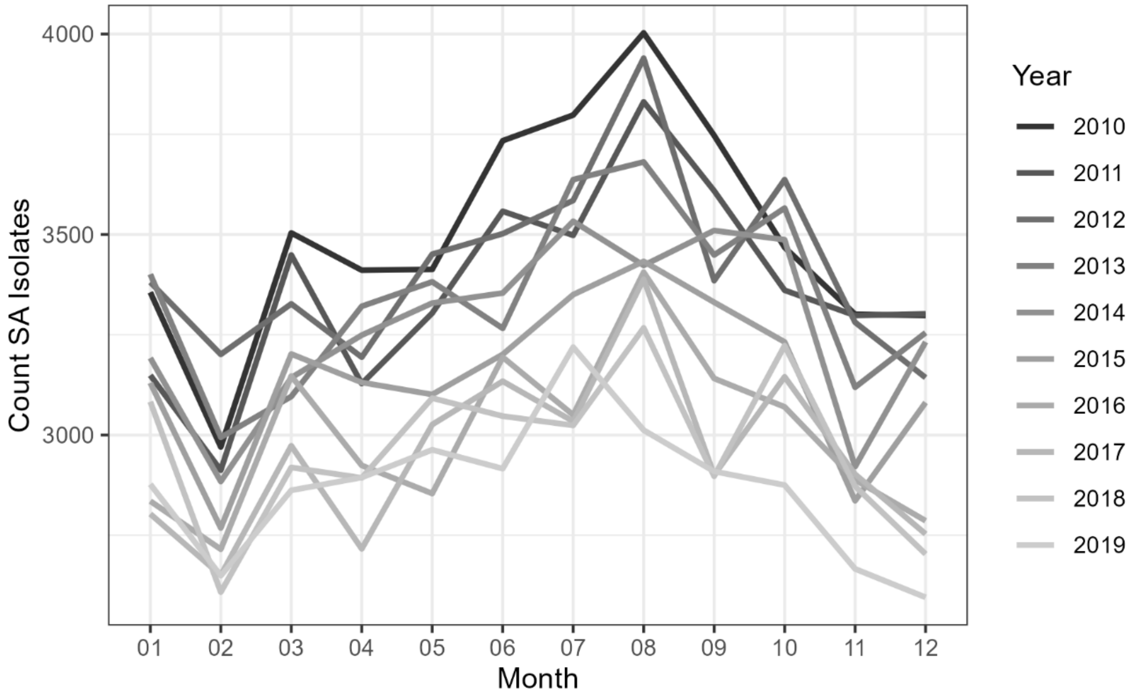

eFigure 1. Count of *S aureus* Isolates by Month, Stratified by Year

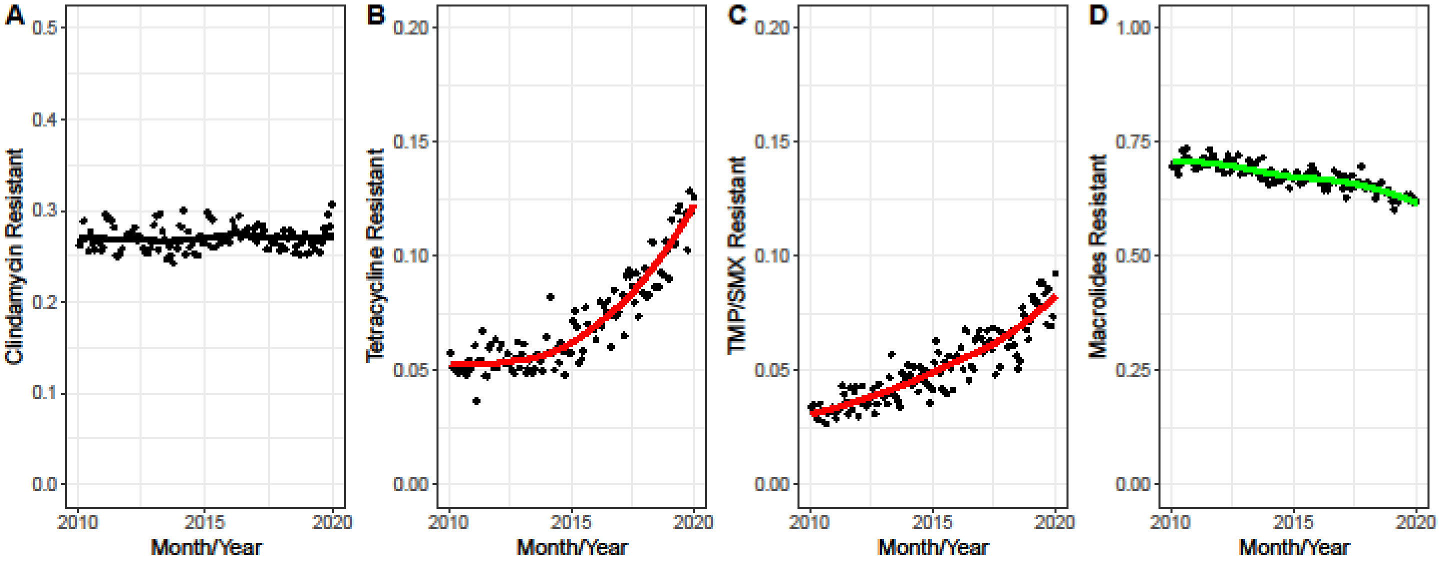

eFigure 2. Prevalence Among MSSA Isolates of Resistance to Clindamycin (A), Tetracyclines (B), Trimethoprim-Sulfamethoxazole (TMP-SMX) (C), and Macrolides (D)

Red lines indicate statistically significant positive trends, green lines indicate statistically significant negative trends, black lines indicate no significant trend.

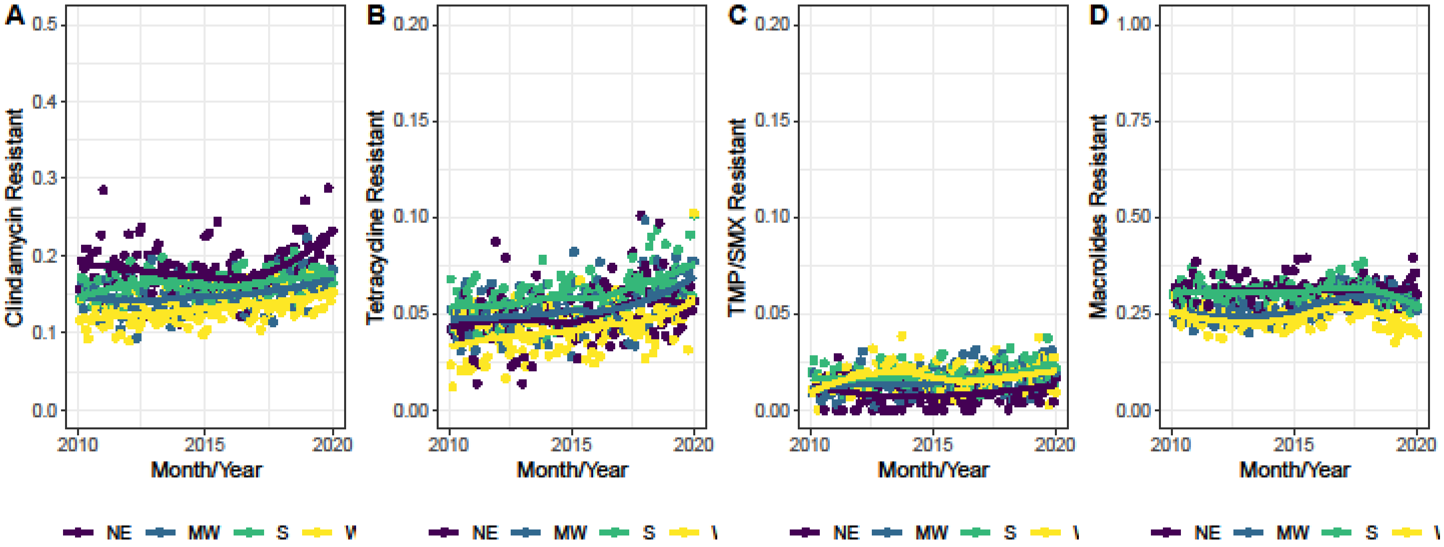

eFigure 3. Regional Prevalence Among MSSA Isolates of Resistance to Clindamycin (A), Tetracyclines (B), Trimethoprim-Sulfamethoxazole (TMP-SMX) (C), and Macrolides (D)

© 2024 Carrel M et al. *JAMA Network Open.*

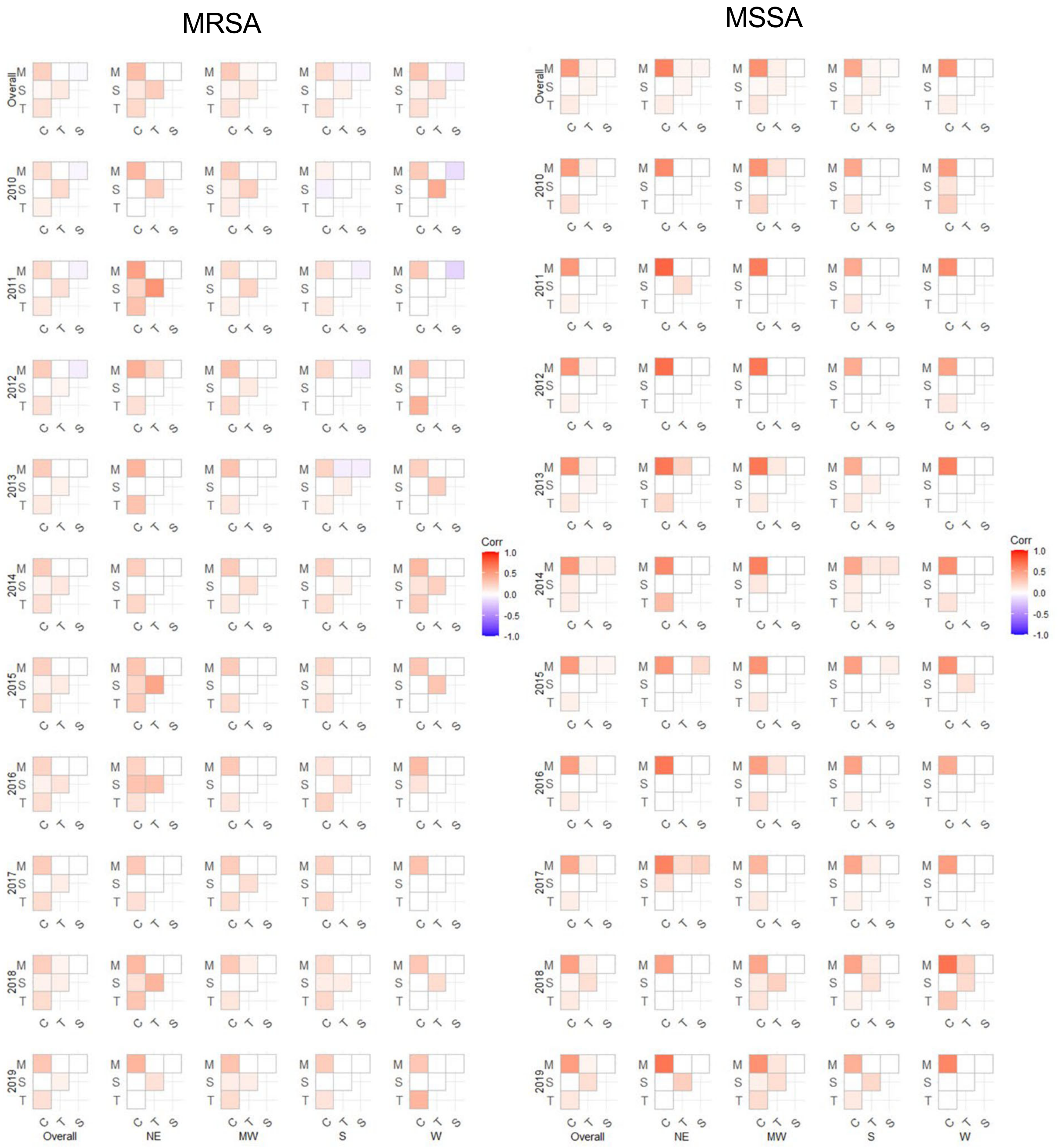

eFigure 4. Pearson Correlation Coefficients in MRSA and MSSA Isolates, Overall and Stratified by Year and Region, for Correlation Between Prevalence Rates of Clindamycin (C), Tetracyclines (T), Trimethoprim-Sulfamethoxazole (S), and Macrolides (M) Resistance

White squares were not statistically significant.
