# Supplementary material for: Antimicrobial Resistance Patterns of Outpatient Staphylococcus aureus Isolates
Source: JAMA Netw Open. 2024 Jun 14;7(6):e2417199. doi: 10.1001/jamanetworkopen.2024.17199 (PMC11179135; doi:10.1001/jamanetworkopen.2024.17199)
Supplement: Supplement 2. — Data Sharing Statement [file jamanetwopen-e2417199-s002.pdf]

Data Sharing Statement

Carrel. Antimicrobial Resistance Patterns of Outpatient Staphylococcus aureus Isolates. *JAMA Netw Open*. Published June 14, 2024. doi:10.1001/jamanetworkopen.2024.17199

Data
Data available: No

Additional Information
Explanation for why data not available: Data is made available from the Veterans Health Administration only by VHA research affiliation.
